# Effect of pituitary‐dependent hypercortisolism on the survival of dogs treated with radiotherapy for pituitary macroadenomas

**DOI:** 10.1111/jvim.16724

**Published:** 2023-05-22

**Authors:** Sofia Rapastella, Simona Morabito, Mellora Sharman, Jérôme Benoit, Luca Schiavo, Joanna Morris, Jane Margaret Dobson, Christopher John Scudder

**Affiliations:** ^1^ Anderson Moores Veterinary Specialists, part of Linnaeus Veterinary Limited, The Granary Winchester UK; ^2^ Anicura Ospedale Veterinario I Portoni Rossi Bologna Italy; ^3^ VetCT ‐ Teleconsulting Hospital, St John's Innovation Centre Cowley Rd Cambridge Cambridge UK; ^4^ Oncovet Villeneuve d'Ascq France; ^5^ University of Cambridge Ringgold standard institution ‐ Veterinary Oncology Cambridge UK; ^6^ University of Glasgow School of Veterinary Medicine Ringgold standard institution ‐ Small Animal Hospital Glasgow UK; ^7^ Department of Veterinary Medicine University of Cambridge, Queen's Veterinary School Hospital Cambridge UK; ^8^ The Royal Veterinary College London UK; ^9^ Southfields Veterinary Specialists Laindon UK

**Keywords:** adenohypophysis, adrenal gland, canine, Cushing, endocrinology, enocrinology, hyperadrenocorticism, PDH, pituitary gland

## Abstract

**Background:**

Radiotherapy (RT) is an effective treatment for dogs presented with neurologic signs caused by pituitary tumors. However, its impact on the outcome of concurrent pituitary‐dependent hypercortisolism (PDH) is controversial.

**Objectives:**

Determine whether dogs with PDH have longer survival after pituitary RT compared with dogs with nonhormonally active pituitary masses and to evaluate whether clinical, imaging, and RT variables affect survival.

**Animals:**

Ninety‐four dogs divided into 2 groups: PDH and non‐PDH, based on the presence of hypercortisolism. Forty‐seven dogs were allocated to the PDH group and 47 to the non‐PDH group.

**Methods:**

Retrospective cohort study in which clinical records of dogs undergoing RT for pituitary macroadenomas between 2008 and 2018 at 5 referral centers were retrospectively evaluated.

**Results:**

Survival was not statistically different between PDH and non‐PDH groups (median survival time [MST], 590 days; 95% confidence interval [CI], 0‐830 days and 738 days; 95% CI, 373‐1103 days, respectively; *P* = .4). A definitive RT protocol was statistically associated with longer survival compared with a palliative protocol (MST 605 vs 262 days, *P* = .05). The only factor statistically associated with survival from multivariate Cox proportional hazard analysis was total radiation dose (Gy) delivered (*P* < .01).

**Conclusions and Clinical Importance:**

No statistical difference in survival was identified between the PDH and non‐PDH groups, and longer survival was associated with higher Gy delivered.

**Abbreviations**
ACTH: adrenocorticotropic hormone
ACTHst: adrenocorticotropic hormone stimulation test
CTV: clinical target volume
CT: computed tomography
DI: diabetes insipidus
DM: diabetes mellitus
eACTH: endogenous ACTH
Gy: Grays
GTV: gross tumor target volume
HAC: hypercortisolism
KCS: keratoconjunctivitis sicca
LDDST: low‐dose dexamethasone suppression test
MST: median survival time
MRI: magnetic resonance imaging
PDH: pituitary‐dependent hypercortisolism
PBR: pituitary‐to‐brain ratio
PTV: planning target volume
PUPD: polyuria and polydipsia
RT: radiotherapy
UCCR: urine cortisol‐to‐creatinine ratio

## 1 INTRODUCTION

Pituitary tumors are considered uncommon in dogs.^1^ However, they represent up to 25% of all intracranial neoplasms in middle‐age and geriatric dogs and are the most common tumors of the sella turcica.^1^, ^2^, ^3^ Dogs with pituitary tumors can present with functional endocrine manifestations, have neurologic signs because of a space‐occupying lesion, or a combination of both.

For functional pituitary tumors, pituitary‐dependent hypercortisolism (PDH) is the most common endocrine disease, resulting from hypersecretion of adrenocorticotropic hormone (ACTH).^4^, ^5^, ^6^ Other hormonally active pituitary tumors are rare.

Neurological abnormalities in dogs with pituitary tumors have been associated with tumor dimension, but neurological signs have been described even when tumors are <10 mm in height.^4^, ^7^ Controversy remains regarding whether neurologic signs relate to the size of the mass alone or the rate of tumor growth.^8^ Endocrine function of pituitary tumors does not appear to be associated with pituitary size, and approximately 50% of dogs with PDH have tumors <3 mm, 30% to 40% have masses between 3 and 10 mm and 10% to 20% have tumors >10 mm in height.^9^, ^10^ In dogs with PDH, only 10% to 30% are reported to have concurrent neurologic signs.^8^, ^11^, ^12^

Regardless of whether pituitary tumors are functional or not, ~50% of dogs develop neurological signs, reflecting altered thalamic, hypothalamic, and optic chiasm function. Clinical signs range from altered mentation and behavior to more complex neurologic abnormalities leading to blindness, ataxia, head tilt, cervical hyperesthesia, cranial nerve deficits, seizures, or a combination of these.^2^, ^5^, ^7^

Adenoma resection is the treatment of choice for PDH in humans^13^ and hypophysectomy has been reported to be successful in dogs.^14^, ^15^ However, extension of the pituitary gland above the sella turcica and increased pituitary size make hypophysectomy in dogs technically challenging, with a high incidence of complications.^15^, ^16^ For this reason, and other financial and long‐term management factors, hypophysectomy is not routinely performed in dogs.

Pituitary radiotherapy (RT) is an alternative strategy to manage neurological signs caused by large pituitary tumors in dogs.^4^, ^5^, ^17^, ^18^, ^19^ It extends survival when compared with medical management or no treatment, and has proven efficacy in decreasing tumor size.^4^, ^17^, ^18^, ^20^ It remains unclear whether pituitary RT decreases hormone secretion or improves clinical control of hypercortisolism (HAC), with most studies suggesting an inconsistent effect.^4^, ^5^, ^16^, ^21^ Few studies, however, have assessed whether or not concurrent PDH affects outcome in dogs treated using RT.

Definitive and palliative RT protocols previously have been associated with different outcomes in dogs with pituitary macroadenoma,^19^ an effect that was not directly associated with ability of the tumor to secrete cortisol.

Our primary aim was to determine whether the presence of PDH affected survival in dogs undergoing pituitary RT. We hypothesized that dogs with pituitary tumors and PDH would have improved survival because the clinical signs of HAC would lead to earlier detection. A second aim was to evaluate clinical, RT, and imaging factors that might affect the outcome.

## 2 MATERIALS AND METHODS

### 2.1 Case selection

In this multicenter, retrospective, cohort, observational study, we recruited dogs presented for investigation of neurologic or endocrine signs, or both, that underwent RT for pituitary macroadenoma at 5 referral centers between January 2008 and January 2018. Pituitary macroadenoma was defined as a tumor localized in the pituitary region with suprasellar extension on brain imaging. No specific numerical pituitary height, volume, or pituitary‐to‐brain ratio (PBR) cut‐off was used to define a pituitary tumor as macroadenoma.

Data were obtained from available medical records, by contacting owners using a written questionnaire or both. Clinical presentation and endocrine test results were reviewed by a single veterinarian at each participating institution. Clinical data collected included signalment, clinical presentation, presence of co‐morbidities, medications administered at the time of diagnosis, and any endocrine test performed. Date and cause of death or euthanasia were recorded, or date of last follow‐up if date of death was not available.

Dogs were divided into 2 groups, a PDH group and a non‐PDH group. Dogs were included in the PDH group if a diagnosis of PDH was made on the basis of appropriate clinical signs and compatible endocrine test results and diagnostic imaging findings. A positive urine cortisol‐to‐creatinine ratio (UCCR) alone was not considered sufficient for the diagnosis of HAC. Dogs were included in the non‐PDH group on the basis of absent signs of HAC or endocrine test results for HAC not compatible with excess cortisol production or both, together with a pituitary mass identified during diagnostic imaging. Endocrine testing to exclude HAC was not a requirement if clinical signs of hypercortisolemia were absent. Dogs were excluded if insufficient information was available to adequately classify dogs into the above groups, or if data regarding RT was incomplete.

The study received ethical approval from the Animal Health Trust Ethical Committee in February 2019.

### 2.2 Imaging

All diagnostic imaging studies were reviewed by a single author (SM), using Horos v4.0.0 RC5. Where available, both pre‐treatment and post‐treatment studies were compared. All studies were evaluated using window parameters identified as ideal for pituitary assessment in post‐contrast computed tomography (CT) or magnetic resonance imaging (MRI) studies.^22^, ^23^ For each imaging study, the following variables were evaluated: imaging modality, pituitary‐to‐brain ratio (PBR) assessed using the equation (height of the pituitary gland/cross‐section area of the brain) multiplied by 100,^22^, ^24^ pituitary height (mm), tumor volume (cm^3^), presence of pituitary cysts, hemorrhage, lateralization of the lesion, presence of peritumoral edema, flattening of the basisphenoid, and presence of hydrocephalus. In each dog, the height and width of the pituitary were measured on the image that contained the largest cross‐section of the gland.

Pituitary volume was calculated using the Horos v4.0.0 RC5 closed polygon tool: the region of interest (ROI) was marked on all transverse slices from the most rostral to the most caudal part of the pituitary mass and the ROI/ROI volume/compute volume tool calculated the volume of the lesion by summing the volumes of the ROIs of each slice.

### 2.3 Radiotherapy

Radiotherapy was prescribed to decrease tumor size, ameliorate neurologic signs associated with the tumor mass effect or both in all cases. The following RT variables were recorded: type of linear accelerator, planning software, RT protocol, frequency, presence of treatment interruptions, Gray (Gy) dose per fraction, total Gy dose, field number and angulation, Gy per field, gross tumor volume (GTV), clinical target volume (CTV), planning target volume (PTV), and occurrence of any acute or delayed adverse effects after RT. An RT protocol was defined as definitive when the treatment was delivered either 5 days/week (Monday to Friday) or on a Monday‐Wednesday‐Friday schedule, depending on clinician preference. An RT protocol was defined as palliative when the treatment was delivered once per week, typically for 4 or 5 doses in total. None of the included cases underwent stereotactic radiotherapy (SRT). The RT variables included in our study followed previously published guidelines,^25^ but much of the data recommended in these guidelines unfortunately was not available retrospectively.

### 2.4 Statistical methods

Statistical analysis was performed using Microsoft Excel 16.46, SPSS Version 28.0.1.1, and GraphPad Prism Version 9.4.1. for Mac. Normality was assessed using histograms and Shapiro‐Wilk tests. Fisher's exact or *χ* ^2^ tests were used to assess proportions in each group. The Kaplan‐Meier method with log rank tests was used to estimate the effect of individual clinical variables, imaging characteristics, and RT variables with survival defined as the difference between the first day of RT treatment and the date of death or last follow‐up. Dogs lost to follow‐up or that were alive at the time of statistical analysis were censored and time between first day of RT and last contact with a veterinarian was used for analysis. Logistic regression was used (Mantel‐Cox log‐rank test) to assess the association of endocrine status, clinical findings, RT, and imaging variables with survival. Variables with *P* < .2 in univariate Cox analysis were used for the multivariate analysis using a backward elimination approach.

## 3 RESULTS

### 3.1 Clinical variables

Ninety‐four dogs met the inclusion criteria, with 47 assigned to the PDH group and 47 to the non‐PDH group.

Endocrine testing had been undertaken in 60/94 (63.8%) dogs (47/47 [100%] in the PDH and 13/47 [27.6%] in the non‐PDH group).

In the PDH group, 36/47 dogs (76.6%) were diagnosed based on an adrenocorticotropic hormone (ACTH) stimulation test (ACTHst) only, 7/47 (14.9%) had a combination of ACTHst and low‐dose dexamethasone suppression test (LDDST), 3/47 (6.4%) had ACTHst and endogenous ACTH (eACTH) assay and 1/47 (2.1%) had ACTHst, LDDST, and eACTH.

Within the non‐PDH group, 7/47 (14.9%) dogs had ACTHst assessment only, 3/47 (6.4%) had ACTHst and LDDST, 1/47 (2.1%) had LDDST only, 1/47 (2.1%) had eACTH only, 1/47 (2.1%) UCCR only, and 34/47 (72.4%) of non‐PDH dogs had no endocrine tests performed.

No significant differences in age or body weight at diagnosis were found between groups, with the mean age in both groups being 8.5 years (*P* = .68), and median body weight being 16.75 kg in the PDH group and 19.2 kg in the non‐PDH group (*P* = .55). The proportion of females and males was 42.6% and 57.4% in both groups (*P* = 1.00).

Crossbreed dogs were most commonly identified within the study population (n = 14), followed by Staffordshire Bull Terrier (n = 11), Greyhound (n = 8), Jack Russell Terrier (n = 8), Labrador Retriever (n = 8), Cocker Spaniel (n = 6), Boxer (n = 6), Cavalier King Charles Spaniel (n = 3), Bichon Frise (n = 3), Border Terrier (n = 3), French Bulldog (n = 3), West Highland White Terrier (n = 2), English Bull Terrier (n = 2), Lurcher (n = 2), and Golden Retriever (n = 2). Other breeds were represented by 1 case only. As previously reported, a high prevalence of terrier breeds (27/94, 28.7%) was observed.

Presenting clinical signs are summarized in Table 1. Neurological signs were reported in 67/94 (71.3%) dogs, 31 (65.9%) in the PDH, and 36 (76.6%) in the non‐PDH group, with abnormal mentation being most common. A complete and detailed description of the neurologic examination was not always available. No difference was found in the proportions of dogs presented with neurological signs in either group (*χ* ^2^[1,94] = 1.23; *P* = .25) nor in the proportion of dogs with other presenting signs.

**TABLE 1:** Presenting clinical signs of the 94 dogs included in our cohort and their prevalence within this population. The most common presenting signs were neurologic abnormalities, followed by polyuria and polydipsia (PUPD) and lethargy.

Clinical sign	Number of dogs	Percentage (%)	PDH	Non‐PDH
Neurologic abnormalities	67	71.3	31	36
Abnormal mentation	24	25.5	10	14
Ataxia	16	17.0	11	5
Behavioral changes	14	14.9	7	7
Circling	10	10.6	7	3
Tremors	10	10.6	7	3
Tonic‐clonic seizures	8	8.5	5	3
Head pressing	7	7.4	4	3
Head tilt	4	4.2	1	3
Pacing	3	3.2	2	1
Partial seizure	2	2.1	1	1
Opisthotonos	1	1.1	1	0
Nystagmus	1	1.1	0	1
Bilateral miosis	1	1.1	0	1
Hyperesthesia	1	1.1	1	0
Temporal amyotrophia	2	2.1	0	2
Trismus	2	2.1	1	1
Facial paralysis	2	2.1	1	1
Blindness	10	10.6	2	8
Lethargy	25	26.6	13	12
PUPD	28	29.7	19	9
Cutaneous abnormalities	4	4.2	3	1
Weight loss	4	4.2	3	1
Collapse	4	4.2	2	2
Exophthalmos	1	1.1	1	0
Dysuria	1	1.1	0	1
Dyschezia	1	1.1	0	1

Lethargy was the second most common presenting sign after neurologic abnormalities and was reported in 25/94 dogs (26.6%), 13 in the PDH (27.6%), and 12 (25.5%) in the non‐PDH group.

Polyuria and polydipsia (PUPD) were described in 28/94 (20.2%) dogs, of which 19 were in the PDH group (40.4%) and 9 in the non‐PDH group (19.1%).

The most frequent medications administered before RT included trilostane in 37/94 (39.3%) dogs, prednisolone in 26/94 (27.7%), levothyroxine in 9/94 (9.6%), analgesics in 9/94 (9.6%), and desmopressin in 6/94 (6.4%).

All 37 dogs that received trilostane before RT were in the PDH group. Of those, the trilostane dose remained unchanged post‐RT in 8, was decreased in 9, and was discontinued in 12 dogs. Two dogs had their trilostane dose increased at subsequent follow‐up, and for 6 dogs it was not possible to determine the post‐RT dose prescribed. The reason for trilostane dose discontinuation was available for 5 dogs, and was attributed to hypoadrenocorticism (n = 2), lethargy and anorexia (n = 2), and relapse of neurologic signs (n = 1). In this last dog, it was explicitly recorded that the decision for trilostane discontinuation was the potential anti‐inflammatory effects of endogenous corticosteroids in decreasing peritumoral edema. The rationale behind dose changes in other cases could not be determined from the medical records.

One dog in the PDH group had not been treated with trilostane before RT, but was prescribed trilostane after RT. Two dogs classified as non‐PDH received trilostane a few months after RT treatment. One of these dogs did not have any reported signs of HAC before RT and no endocrine tests were performed before RT. The remaining dog had PUPD at the time of pituitary macroadenoma diagnosis but both ACTHst and LDDST were not consistent with HAC. The rationale for starting trilostane in these 2 dogs was not available.

Fifteen of 94 dogs (15.9%) had concurrent endocrinopathies, 9/15 in the PDH and 6/15 in the non‐PDH group. Of these, 2 dogs had concurrent diabetes mellitus (DM): 1 in the PDH and 1 in the non‐PDH group. Six dogs had a previous diagnosis of diabetes insipidus (DI), 2 of which were in the PDH and 4 in the non‐PDH group. Fifteen dogs had a prior diagnosis of hypothyroidism, 6 in the PDH, and 9 in the non‐PDH group. One dog in the non‐PDH group was diagnosed with hypersomatotropism, concurrent DM, and hypothyroidism.^26^

No difference was found between the 2 groups in the proportions of dogs with concurrent endocrinopathies or in those receiving medications. The endocrine testing leading to diagnosis of the concurrent endocrinopathies was not available for review.

### 3.2 Imaging

Pre‐radiotherapy images were available for review in 76/94 (88.3%) dogs, with diagnostic imaging reports available for the remaining 18. In 49 of these, a macroadenoma had been diagnosed by CT, 35 by MRI and 10 dogs had both CT and MRI imaging before RT. Whenever dogs underwent both imaging modalities, CT was used for RT planning. The type of pre‐RT imaging did not have a statistically significant effect on overall survival (log rank *P* = .78). No significant difference was observed in any of the imaging variables between the PDH and non‐PDH groups (Table 2). The reason for pituitary imaging could not be determined retrospectively.

**TABLE 2:** Different imaging variables (median) in both PDH and PDH groups.

Imaging characteristics	PDH (n = 39)	Non‐PDH (n = 37)	*P* value
Pituitary height (mm)	16.4 (range 6.4‐26.2)	16.3 (range 8.7‐26.4)	.6
PBR	1.01 (range 0.08‐20)	0.98 (range 0.54‐1.51)	.52
Pituitary volume (cm^3^)	2.46 (range 0.14‐7.31)	2.11 (range 0.47‐2.63)	.78

*Note*: Pre‐radiotherapy imaging was available for review in 76/94 dogs, 39 in the PDH and 37 in the non‐PDH group.

Pituitary tumor imaging characteristics (eg, lateralization, hemorrhage, cysts), consequences (e.g., peritumoral edema, flattening of the basisphenoid), or lateralization are summarized in Table 3.

**TABLE 3:** Pituitary imaging characteristics detected in the CT and MRI images available in our study population.

Imaging characteristics	Number of dogs on which the parameter could be evaluated	Number of dogs in which the abnormality was present	PDH	Non‐PDH
Hydrocephalus	51	9	7	2
Flattening of the basisphenoid	51	46	23	23
Peritumoral edema	41	23	12	11
Hemorrhage	45	25	14	11
Pituitary cyst	44	13	6	7
Tumor lateralization	62			
Symmetrical		51	31	20
Right‐sided		7	5	2
Left‐sided		4	1	3

*Note*: Retrospective evaluation of all abnormalities was attempted for each dog and each imaging modality; however, each abnormality could be evaluated only in a subgroup of patients based on the imaging technique performed and on the available slices, windows, and sequences.

### 3.3 Radiotherapy

Eighty dogs (85.1%) underwent a definitive treatment protocol, of which 41 were in the PDH and 39 in the non‐PDH group. Of these, 14 had a Monday‐Wednesday‐Friday protocol (10 in the PDH group, 4 in the non‐PDH group), whereas 66 had a Monday‐to‐Friday protocol (31 in the PDH group, 35 in the non‐PDH group). Fourteen dogs (14.9%) underwent palliative RT, of which 6 were in the PDH and 8 in the non‐PDH group. No significant difference was found in any of the RT protocols or variables between the 2 groups (Table 4). No significant difference was found in the proportions of dogs undergoing palliative or definitive RT, between RT machines (*χ* ^2^[4,89] = 5.7; *P* = .27), or in the proportions of PDH or non‐PDH dogs between machines (*χ* ^2^[4,89] = 6.23, *P* = .18), or in pituitary volume treated by each machine (*P* = .61). Additional details on RT protocol, schedule, and doses are summarized in the Table S1.

**TABLE 4:** Distribution of RT variables (reported as median and range) among the PDH and non‐PDH groups, with associated *P* value.

Parameters	PDH	Non‐PDH	*P* value
Number of RT fractions	10.6 (range 3‐16)	10.9 (range 4‐16)	.36
Time between diagnosis and RT (days)	12.8 (range 0‐212)	11.4 (range 0‐160)	.16
Total radiation dose (Gy)	43.5 (range 12‐50)	44.85 (range 24‐50)	.88
RT protocol (palliative/definitive)	40 D 8 P	41 D 7 P	.77

*Note*: No significant difference in number of RT fractions, time interval between diagnosis and RT, total radiation dose, or type of radiotherapy protocol were found between the two groups.

Abbreviations: D, definitive RT; P, palliative RT.

Median time before initiation of RT treatment after pituitary mass diagnosis was 12 days (range, 3‐390 days) and no significant difference was found between PDH and non‐PDH groups (*P* = .16). The number of fractions prescribed was 5 in all dogs undergoing the palliative protocol, and median was 10 (range, 9‐20) for the definitive protocol. The number of fractions delivered ranged from 1 to 5 in the palliative group, with 2 dogs in the non‐PDH group having an incomplete treatment. In the definitive protocol, the number of fractions administered ranged from 3 to 20, with 4 dogs having incomplete treatment (2 in the PDH group and 2 in the non‐PDH group). Median total radiation dose delivered for dogs undergoing palliative RT was 37 Gy (range, 29‐48) and 45 Gy (range, 12‐50) in dogs undergoing definitive RT. Median total RT dose was 43.5 Gy (range, 12‐50) in the PDH group and 44.85 Gy (range, 24‐50) in the non‐PDH group.

Twenty‐five dogs (26.6%) experienced adverse RT effects. Twenty dogs, all undergoing definitive RT, had acute adverse RT effects, occurring hours or days after RT treatment. Five dogs experienced delayed adverse RT effects, occurring weeks to months after RT, of which 2 dogs received definitive and 3 dogs had received palliative RT. Adverse RT effects and their frequency are listed in Table 5. Most common was deterioration of neurological signs. No significant difference was found in the proportions of patients experiencing adverse RT effects between groups (Fisher's exact *P* = 1.00).

**TABLE 5:** Frequency of radiotherapy adverse effects reported in the study population (94 dogs).

RT side effect	Number of dogs	Percentage (%)	PDH	Non‐PDH
Deterioration of neurological signs	7	7.4	3	4
Otitis externa, dermatitis, mucositis	6	6.4	3	3
Gastrointestinal signs	5	5.3	3	2
Lethargy	4	4.3	1	3
Ocular signs (KCS, ulceration)	3	3.2	3	0
Acquired endocrinopathies (Diabetes insipidus, hypothyroidism)	1	1.1	0	1

Radiotherapy protocol interruptions occurred in 6/94 (6.3%) dogs, because of vomiting (n = 1), abscess development (n = 1), lethargy (n = 1), and the linear accelerator being out of order (n = 3). The RT protocol was not completed in 7/94 dogs (7.4%) because of deterioration of neurological signs (n = 5), euthanasia (n = 1), or severe vomiting (n = 1).

The model and brand of linear accelerator used was available for 89/94 dogs (94.7%), of which 36 were treated using *Varian* 2100C (19 in the PDH group and 17 in the non‐PDH group), 30 using *Varian* 600C (12 in the PDH group and 18 in the non‐PDH group), 12 with *Elekta* Precise (5 in the PDH group and 7 in the non‐PDH group), 10 using *Siemens Oncor Impression* Plus (8 in the PDH group and 2 in the non‐PDH group), and only 1 dog was treated using *Dynaray* (in the PDH group).

The software used for RT planning was available for 90/94 dogs (95.7%): Addenbrooke's Radiotherapy Planning System (ARPS) was used in 37 (20 in the PDH group and 17 in the non‐PDH group), *Prowess* v 4.71, v 5.1 or v 5.51 in 10 (7 in the PDH group and 3 in the non‐PDH group), and Oncentra (*Elekta*) in 43 dogs (19 in the PDH group and 14 in the non‐PDH group).

### 3.4 Post‐treatment imaging

Follow‐up images after RT were available for only 7/94 (7.4%) dogs, 4 in the PDH group and 3 in the non‐PDH group. The interval between RT and repeated brain imaging ranged from 2 to 22 months. Tumor volume reduction and decreased PBR were documented in all dogs in which post‐RT imaging was available. One dog had 4 CT studies post‐RT, which showed progressive decrease in PBR and tumor volume over a period of 13 months (PBR from 0.66 to 0.36; tumor volume from 0.86 to 0.27 cm^3^). The same dog experienced relapse of neurologic signs 22 months after RT, and repeated CT at this time showed increased PBR and pituitary volume (0.48 and 0.49 cm^3^, respectively). It was not possible for the other cases to determine retrospectively whether repeated imaging was performed during routine re‐assessments or because of progression of clinical signs.

### 3.5 Outcome

**FIGURE 1:**
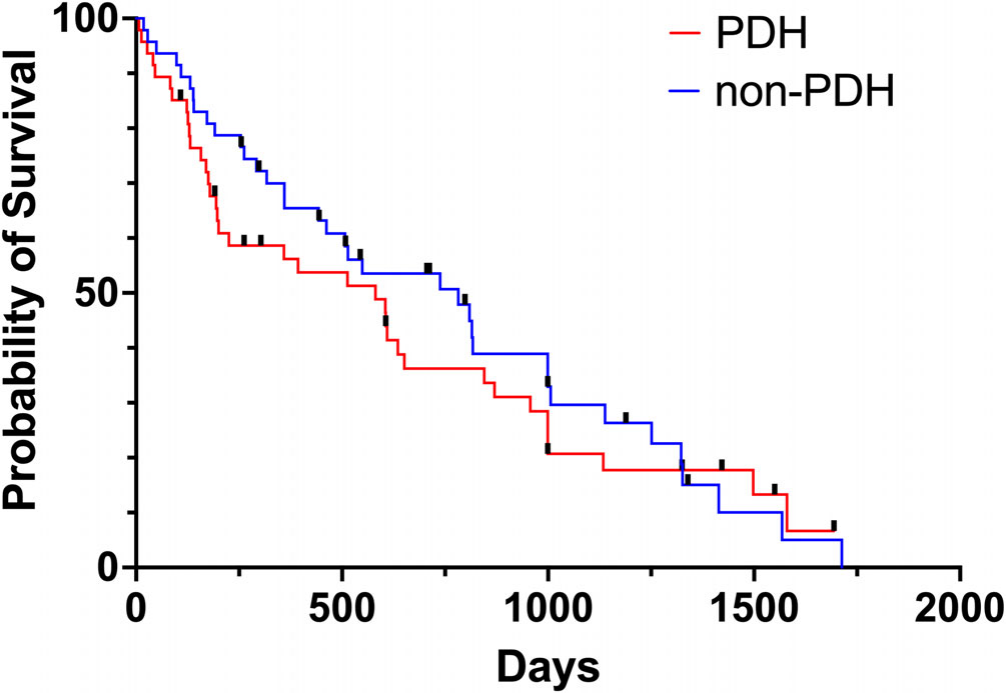
Kaplan‐Meier curve comparing MST of dogs in the PDH group (in red) and non‐PDH group (in blue). The difference in outcome between the two groups was not statistically significant (590 days in PDH group, 738 days in non‐PDH group, *P* = .4). The black perpendicular lines represent censored dogs.

Overall median survival time (MST) for all dogs was 514 days (95% CI, 351‐677). No significant difference was found in survival between dogs in the PDH and non‐PDH groups (MST, 590; 95% CI, 0‐830 days and MST 738; 95% CI, 373‐1103 days, respectively; *P* = .4; Figure 1).

No significant difference was found in survival between PDH and non‐PDH dogs that received definitive or palliative RT (MST of dogs treated with definitive RT and that had PDH was 513 [95% CI, 44‐982] days and without PDH was 809 [95% CI, 464‐1154] days, and MST of dogs treated with palliative RT and that had PDH was 200 [95% CI, 177‐223] and without PDH was 360 [95% CI, 150‐570] days; *P* = .53).

Cause of death was available in 60/94 dogs (63.8%). Deterioration or relapse of neurologic signs was the most common cause (44/60 [73.3%] dogs). Other causes unrelated to pituitary neoplasia are listed in Table 6. Twenty dogs in the PDH group and 24 dogs in the non‐PDH group were euthanized because of worsening neurological signs. Necropsy was not performed in any of the included dogs.

**TABLE 6:** Causes of death or euthanasia on the 60 dogs for which this information was available.

Cause of death	Number of dogs	Percentage (%)	PDH	Non‐PDH
Deterioration of neurological signs	44	73.3	20	24
Heart failure	2	3.3	1	1
Other tumors	5	8.4	3	2
Gastric dilation and volvulus	2	3.3	2	0
Discomfort	3	5.0	1	2
Anorexia	2	3.3	1	1
Renal disease	1	1.7	1	0
Glaucoma	1	1.7	1	0

**FIGURE 2:**
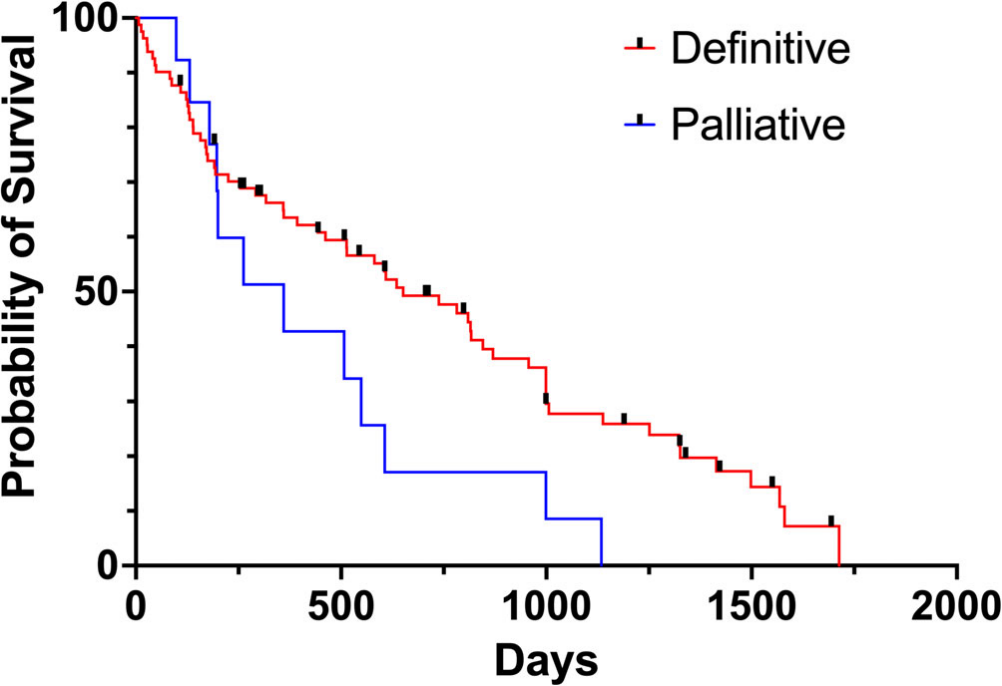
Kaplan‐Meier curve comparing MST of dogs undergoing definitive (in red) and palliative (in blue) radiotherapy. The difference in outcome between the two group was statistically significant (605 days in definitive treatment, 262 days in palliative treatment, *P* = .05). The black perpendicular lines represent censored dogs.

A significant (*P* = .05) difference in MST was found between the definitive protocol group (MST, 605 days) and the palliative protocol group (MST, 262 days; Figure 2). The 2‐ and 3‐year survival fraction of dogs undergoing definitive RT was 32% and 19%, respectively, and for palliative RT was 9% for both 2‐ and 3‐year survival. Among the dogs undergoing definitive RT, no significant difference was found in MST between those treated Monday‐to‐Friday and those treated Monday‐Wednesday‐Friday (*P* = .25).

### 3.6 Multivariate analysis

The univariate Cox analysis variables that met the inclusion criteria for the multivariate analysis (*P* < .2) are presented in Table 7. In multivariate Cox hazard analysis, the only significant variable was total Gy delivered, with higher Gy associated with longer survival (hazard ratio [HR] for death, 0.905; 95% CI, 0.855‐0.958; *P* < .01).

**TABLE 7:** Variables associated with survival with *P* < .2 at univariate Cox regression analysis.

Variable	Hazard ratio	95% CI for hazard ratio		*P* value
		Lower	Upper	
Total Gray	0.926	0.886	0.969	<.001
Age (years)	1.127	1.003	1.268	.04
Definitive vs palliative	1.935	0.995	3.766	.05
Time to radiotherapy	1.003	0.999	1.007	.1

## 4 DISCUSSION

Our main aim was to evaluate differences in outcome between dogs with and without PDH undergoing RT for a pituitary mass and to determine if any factors in signalment, clinical signs, imaging modalities, or RT protocol could predict an effect on survival. Contrary to our hypothesis, no significant difference in MST between dogs with and without PDH was found.

This lack of difference could have been secondary to selection bias, because pituitary RT is typically aimed at achieving tumor shrinkage and alleviation of neurological signs independently from hormonal status, which is consistent with the high proportion of dogs with neurological signs (71.3%) in this cohort. The retrospective nature of our study made it difficult to assess neurological examination findings or severity of neurological presentation. Furthermore, presence of HAC‐associated systemic signs, complications, and costs associated with trilostane treatment might influence owners' perception of quality of life and might have shortened survival in the PDH group.

Our data adds to the conflicting veterinary literature on the association between hormonal status and survival of dogs with pituitary macroadenomas, using both fractionated RT and stereotactic RT protocols. In agreement with our findings, a previous study^19^ described survival as not being affected by hypercortisolemia in dogs with pituitary tumors treated with fractionated RT. However, another study described nonfunctional tumors having worse outcome compared with dogs with HAC when fractionated RT was used.^4^ Yet another study performed by the same research group, and describing the long‐term outcome of dogs treated with stereotactic as opposed to fractionated RT, found that dogs with nonfunctional masses had longer survival than those with HAC.^27^ Interestingly, the dogs with nonfunctional tumors had longer survival than thus far reported for traditional fractionated radiation and the authors hypothesized that corticotrophs of functional tumors may be more sensitive to fractioned than stereotactic RT.

In the previous study,^4^ however, dogs with tumors as small as 3 mm were included if they presented with appropriate neurologic signs that could be attributed to a pituitary location, rather than macroadenomas alone. Further complicating comparisons in the veterinary literature, the term macroadenoma has been used arbitrarily. In humans, it is defined as a pituitary mass with diameter >10 mm. This cut‐off is often also used in dogs, but in various other studies, a tumor >5 mm is considered sufficient to disrupt pituitary anatomy and is therefore used to define a macroadenoma.^28^, ^29^ Other authors define macroadenomas as pituitary tumors with suprasellar extension (which is the definition used in our study) because numerical cut‐offs do not take into account variation in size of the brain in different‐sized breeds.^24^

The PBR has been described as the variable having the highest discriminatory power in distinguishing enlarged from nonenlarged pituitary glands.^24^ All dogs included in our cohort had a PBR >0.31 (range, 0.35‐2), the cut‐off used for defining a pituitary gland as enlarged.^24^

In our study, tumor dimensions (height, volume, PBR) were not associated with survival. This finding contradicts another study of dogs with pituitary masses treated with RT, in which those with smaller tumors (calculated as PBR or area of tumor to area of brain) lived longer than those with larger tumors.^4^ This difference might be attributed to type II statistical error as a result of the smaller number of dogs included (n = 19) or to the different definition of macroadenoma.^4^ Although tumor size at diagnosis did not differ between groups in our study, trilostane treatment might cause a progressive increase in tumor size because of lack of adrenal feedback over time, a condition known in humans as Nelson's syndrome.^11^, ^30^, ^31^, ^32^, ^33^ This potential increase in pituitary size in PDH dogs would counter shrinkage resulting from RT and might account for earlier euthanasia or death because of recurrent neurological signs.

In our study, the finding that definitive RT protocols were associated with prolonged survival is not surprising. Definitive RT protocols allow a higher number of fractions at shorter intervals, maximizing cell repair, redistribution, reoxygenation, and repopulation, and allowing a larger therapeutic window between the minimal tumor control dose and the maximum tolerable dose to the healthy surrounding tissues. On the other hand, definitive protocols are intensive and might be more likely to be recommended in stable patients, thus resulting in a selection bias.

To our knowledge, previous studies have not evaluated how differences in total Gy administered impacts survival in pituitary RT. However, comparing the veterinary literature available, higher total delivered RT dose seems associated with a longer MST (Table 8). A previous study used a definitive protocol (total dose of 48 Gy) and found a MST of 1405 days, compared with untreated controls (MST 359 days; *P* = .004).^4^ Another study described 12 dogs that underwent definitive RT using a total 36 Gy dose, and reported a MST of 539 days.^18^ In another study, a significant difference was identified when 38 Gy was administered as either a palliative or definitive RT protocol with an MST of 182 vs 961 days between these groups (*P* = .01).^19^ Administering higher radiation doses might raise concerns for more frequent adverse effects, but in our study adverse effects were not severe enough to impact outcome. This finding might reflect a relatively high use of peri‐RT corticosteroids in our study population.

**TABLE 8:** Median survival times (MST in days) of dogs undergoing pituitary radiotherapy in the recent literature.

	Definitive MST (days)	Number of dogs (definitive)	Palliative MST (days)	Number of dogs (palliative)	*P* value	Total dose (Gy)
Rapastella et al	605	80	262	14	.05	12‐50 for definitive, 29‐48 for palliative
Kent et al^3^	1405	19	NA	NA	—	48
Marcinowska et al^19^	961	12	182	12	.01	38
De Fornel et al^18^	539	12	NA	NA	—	36

In the included cohort, a decrease in PDH signs, or a consistent decrease in trilostane dosage in those dogs receiving this drug, could not be determined because of the retrospective nature of the study. Endocrine testing was not performed at regular intervals after RT, and limited data were available to assess the endocrine response to RT. Although some dogs experienced a decrease in trilostane dosage, or discontinuation, the rationale for this adjustment was not always available, and these changes could have been secondary to resolution of HAC signs, hypoadrenocorticism, concurrent diseases, owner finances, or relapse of neurologic abnormalities.

In view of these considerations, a prospective study design might help resolve existing conflict in the veterinary literature about the role of RT in improving clinical signs associated with HAC. In people, more aggressive RT protocols are required to overcome hormonal production compared with doses used for mass reduction.^34^ Another consideration might be that a difference in sensitivity to RT exists, depending upon the tumor type present (i.e., carcinoma vs adenoma) or secretory cell origin (corticotroph vs somatotroph). One dog in the non‐PDH group in our study with multiple endocrinopathies (DM, hypersomatotropism, hypothyroidism), and that underwent treatment using both RT and pasireotide, achieved resolution of acromegaly as well as remission of DM.^26^

A surprisingly low proportion of dogs in the PDH group had PUPD (19/47, 40.4%). Dogs in this group most likely were diagnosed with HAC and started on medical treatment before being referred for RT, and their HAC signs, including PUPD, might have been controlled at the time of presentation for RT. It is also possible that PDH dogs might have displayed other signs (e.g., neurologic) that were considered more severe than PUPD and that, despite the presence of PUPD, this concern was not explicitly included in the clinical notes.

Our study had several limitations, mainly because of its retrospective nature across multiple hospitals. Primarily this design meant there was a lack of standardization of diagnostic testing and RT protocols, including endocrine testing and post‐RT monitoring. Despite our efforts, a misclassification of dogs between the 2 groups is possible because of secondhand evaluation of the content and detail of the clinical notes as well as the intrinsic limitations of endocrine testing. As in similar studies on pituitary RT, no pituitary histology was available. Different histological characteristics may affect responsiveness to treatment, but adenohypophyseal hyperplasia and adenoma are the most common pituitary diagnoses in dogs, with other sellar neoplasms being rare.^35^, ^36^, ^37^ Furthermore, the definition of pituitary carcinoma or adenoma is based on the presence or absence of metastatic disease, and no dog in this study had known metastases.

Considering these limitations and current gaps in the veterinary literature, prospective, multicenter studies are needed, with standardized diagnostic testing, RT protocols and planning, and appropriate follow‐up, to assess the clinicopathologic effects of RT on pituitary secretory properties.

In conclusion, we did not identify clinical characteristics of pituitary tumors associated with survival. However, RT protocol did affect outcome in our cohort, independently of patient hormonal status, and a higher total radiation dose (Gy) was associated with longer survival.

## CONFLICT OF INTEREST DECLARATION

Authors declare no conflict of interest.

## OFF‐LABEL ANTIMICROBIAL DECLARATION

Authors declare no off‐label use of antimicrobials.

## INSTITUTIONAL ANIMAL CARE AND USE COMMITTEE (IACUC) OR OTHER APPROVAL DECLARATION

Approved by Animal Health Trust Ethical Committee, project number 39‐2018.

## HUMAN ETHICS APPROVAL DECLARATION

Authors declare human ethics approval was not needed for this study.

## Supporting information


**Table S1.** Radiotherapy planning, doses and delivery parameters.
