# Supplementary material for: Effect of pituitary‐dependent hypercortisolism on the survival of dogs treated with radiotherapy for pituitary macroadenomas
Source: J Vet Intern Med. 2023 May 22;37(4):1331–40. doi: 10.1111/jvim.16724 (PMC10365051; doi:10.1111/jvim.16724)
Supplement: Supplementary file 1 — Table S1. Radiotherapy planning, doses and delivery parameters. [file JVIM-37-1331-s001.pdf]

Table 1

	RT protocol	RT schedule	Number of fractions delivered	Dose/fraction (Gy)	Total delivered dose (Gy)	Dose (cGy)/field	RT fields number and direction	Patient position	Linear accelerator brand	Planning software
1	Definitive	Mon-Fri	10	4	40	133	3 (1 anterior and 2 laterals)	Sternal	Varian 2100C, 6Mv	ARPS
2	Definitive	Mon-Fri	10	3.8	38	162 (anterior) 109 (lateral)	3 (1 anterior and 2 laterals)	sternal	varian 2100C, 6Mv	ARPS
3	Definitive	Mon-Fri	10	4	40	162	3 (1 anterior and 2 laterals)	sternal	varian 2100C, 6Mv	ARPS
4	Definitive	Mon-Fri	10	4	40	142 (anterior) 129 (lateral)	3 (1 anterior and 2 laterals)	sternal	varian 2100C, 6Mv	ARPS
5	Definitive	Mon-Fri	10	3.8	38	164 (anterior) 108 (lateral)	3 (1 anterior and 2 laterals)	sternal	Dynaray 4, 4Mv	ARPS
6	Definitive	Mon-Fri	10	4	40	148 (anterior) 165 (lateral)	3 (1 anterior and 2 laterals)	sternal	Varian 2100C, 6Mv	ARPS
7	Definitive	Mon-Fri	12	4	48	112 (DV) 145 (left) 141 (right)	3 (1 dorsal and 2 laterals)	sternal	varian 2100C, 6Mv	ARPS
8	Definitive	Mon-Fri	12	4	48	133	3 (1 anterior and 2 laterals)	sternal	varian 2100C, 6Mv	ARPS
9	Definitive	Mon-Fri	6	4	24	123 (anterior) 176 (lateral)	3 (1 anterior and 2 laterals)	sternal	Varian 2100C, 6Mv	ARPS
10	Definitive	Mon-Fri	10	3.7	37	174 (anterior) 98 (lateral)	3 (1 anterior and 2 laterals)	sternal	varian 2100C, 6Mv	ARPS
11	Definitive	Mon-Fri	10	3.7	37	154 (anterior) 108 (lateral)	3 (1 anterior and 2 laterals)	sternal	Varian 2100C, 6Mv	ARPS
12	Definitive	Mon-Fri	10	3.8	38	160 (anterior) 134 (lateral).	3 (1 anterior and 2 laterals)	sternal	Varian 2100C, 6Mv	ARPS
13	Definitive	Mon-Fri	10	4	40	160	3 (1 dorsal and 2 laterals)	sternal	Varian 2100C, 6Mv	ARPS
14	Palliative	Once weekly	5	5Gy for 1 dose, 8Gy for 4 doses	37	169 (dorsal) - 164 (R) 166 (L) during the first session; 271 (dorsal) 262 (R) 266 (L) second session	3 (1 dorsal and 2 laterals)	sternal	Varian 2100C, 6Mv	ARPS
15	Definitive	Mon-Fri	10	3.8	38	163 (dorsal) 108 (lateral)	3 (1 dorsal and 2 laterals)	sternal	Varian 2100C, 6Mv	ARPS
16	Palliative	Once weekly	4	5Gy for 1 dose, 8Gy for 3 doses	29	212 (dorsal) 204 (lateral) for 1 dose - 340 (dorsal) 226 (R) 233 (L) for 3	3 (1 dorsal and 2 laterals)	sternal	Varian 2100C, 6Mv	ARPS
17	Definitive	Mon-Fri	9	3.7	33.3	126 (dorsal) 122 (lateral)	3 (1 dorsal and 2 laterals)	sternal	Varian 2100C, 6Mv	ARPS
18	Palliative	Once weekly	5	5Gy for 1 dose, 8Gy for 4 doses	37	170 (dorsal) 165 (lateral) for 1 dose - 272 (dorsal) 263 (lateral) for 4	3 (1 dorsal and 2 laterals)	sternal	Varian 2100C, 6Mv	ARPS
19	Definitive	Mon-Fri	10	3.8	38	161 (dorsal) 107 (R) 111(L)	3 (1 dorsal and 2 laterals)	sternal	Varian 2100C, 6Mv	ARPS
20	Palliative	Once weekly	5	5Gy for 1 dose, 8Gy for 4 doses	37	213 (dorsal) 141 (R) 144 (L) for 1 dose - 342 (dorsal) 226 (R) 230 (L) for	3 (1 dorsal and 2 laterals)	sternal	Varian 2100C, 6Mv	ARPS
21	Palliative	Once weekly	5	5Gy for 1 dose, 8Gy for 4 doses	37	170 (dorsal) 166 (R) 163 (L) for 1 dose - 272 (dorsal) 265 (R) 261 (L) for	3 (1 dorsal and 2 laterals)	sternal	Varian 2100C, 6Mv	ARPS
22	palliative	Once weekly	5	5Gy for 1 dose, 8Gy for 4 doses	37	211 (dorsal) 142 (R) 145 (L) for 1 dose - 339 (dorsal) 227 (R) 233 (L) for	3 (1 anterior and 2 laterals)	sternal	varian 2100C, 6Mv	ARPS
23	Definitive	Once weekly	10	3.75	37.5	156 (dorsal) 109 (lateral)	3 (1 dorsal and 2 laterals)	sternal	Varian 2100C, 6Mv	ARPS
24	Definitive	Mon-Fri	10	4	40	173 (anterior) 122 (R) 114 (L).	3 (1 anterior and 2 laterals)	sternal	Varian 2100C, 6Mv	ARPS
25	Palliative	Once weekly	5	5Gy for 1 dose, 8Gy for 4 doses	37	176 (dorsal) 162 (lateral) for 1 dose - 281 (dorsal) 260 (R) 257 (L) for 4	3 (1 dorsal and 2 laterals)	sternal	Varian 2100C, 6Mv	ARPS
26	Palliative	Once weekly	5	5Gy for 1 dose, 8Gy for 4 doses	37	133 (dorsal) 185 (R) 181(L) for 1 dose - 213 (dorsal) 296 (R) 289 (L) for	3 (1 dorsal and 2 laterals)	sternal	Varian 2100C, 6Mv	ARPS
27	Definitive	Mon-Fri	10	3.7	37	160 (posterior) 105 (R) 104 (L)	3 (posterior and 2 laterals)	sternal	Varian 2100C, 6Mv	aRPS
28	Definitive	Mon-Fri	10	4	40	168 (anterior) 117 (R) 113 (L)	3 (1 anterior and 2 laterals)	sternal	Varian 2100C, 6Mv	ARPS
29	Definitive	Mon-Fri	10	3.8	38	159 (posterior) 111 (R) 108 (L)	3 (1 posterior and 2 laterals)	sternal	Varian 2100C, 6Mv	ARPS
30	Definitive	Mon-Fri	10	4	40	85 (dorsal) 157 (R) 158 (L)	3 (1 dorsal and 2 laterals)	sternal	Varian 2100C, 6Mv	ARPS
31	Definitive	Mon-Fri	10	4	40	137 (dorsal) 131 (lateral)	3 (1 dorsal and 2 laterals)	sternal	Varian 2100C, 6Mv	ARPS
32	Definitive	Mon-Fri	3	4	12	135 (dorsal) 131 (L) 132 (R)	3 (1 dorsal and 2 laterals)	sternal	Varian 2100C, 6Mv	ARPS
33	Definitive	Mon-Fri	10	4	40	142 (dorsal) 127 (R) 130 (L)	3 (1 dorsal and 2 laterals)	sternal	Varian 2100C, 6Mv	ARPS
34	Definitive	Mon-Fri	10	4	40	176	3 (1 dorsal and 2 laterals)	sternal	varian 2100C, 6Mv	ARPS
35	Definitive	Mon-Fri	10	3.8	38	162 (anterior) 108 (R) 109 (L)	3 (1 anterior and 2 laterals)	sternal	Varian 2100C, 6Mv	ARPS
36	Definitive	Mon-Fri	9	3.8Gy for 6 doses, 4.5Gy for 3 doses	36	160 (posterior) 110 (lateral) for 6 doses - 190 (posterior) 130 (lateral) for 3	3 (1 posterior and 2 laterals)	sternal	Varian 2100C, 6Mv	ARPS
37	Definitive	Mon-Fri	20	NA	50	250	4 (1 dorsal, 1 ventral, 2 laterals)	NA	NA	NA
38	Definitive	Mon-Fri	20	NA	50	250	4 (1 dorsal, 1 ventral, 2 laterals)	NA	NA	NA
39	Definitive	Mon-Fri	20	NA	50	250	4 (1 dorsal, 1 ventral, 2 laterals)	NA	NA	NA
40	Definitive	Mon-Fri	20	NA	50	250	4 (1 dorsal, 1 ventral, 2 laterals)	NA	NA	NA
41	Definitive	Mon-Fri	20	NA	50	250	4 (1 dorsal, 1 ventral, 2 lateral)	NA	NA	NA
42	Definitive	Mon-Wed-Fri	12	4	48	133	3 (1 dorsal, 2 laterals)	sternal	Siemens Oncor Impression Plus	Prowess v 4.71
43	Definitive	Mon-Wed-Fri	12	4	48	133	3 (1 dorsal, 2 laterals)	sternal	Siemens Oncor Impression Plus	Prowess v 4.71
44	Definitive	Mon-Wed-Fri	12	4	48	133	3 (1 dorsal, 2 laterals)	sternal	Siemens Oncor Impression Plus	Prowess v 4.71
45	Definitive	Mon-Wed-Fri	12	4	48	133	3 (1 dorsal, 2 laterals)	sternal	Siemens Oncor Impression Plus	Prowess v 4.71
46	Definitive	Mon-Fri	16	3	48	100	3 (1 dorsal, 2 laterals)	sternal	Siemens Oncor Impression Plus	Prowess v4.71
47	Definitive	Mon-Fri	16	3	48	100	3 (1 dorsal, 2 laterals)	sternal	Siemens Oncor Impression Plus	Prowess v 4.71
48	Definitive	Mon-Fri	16	3	48	100	3 (1 dorsal, 2 laterals)	sternal	Siemens Oncor Impression Plus	Prowess v5.10
49	Definitive	Mon-Fri	16	3	48	100	3 (1 dorsal, 2 laterals)	sternal	Siemens Oncor Impression Plus	Prowess v5.10
50	Definitive	Mon-Fri	16	3	48	100	3 (1 dorsal, 2 laterals)	sternal	Siemens Oncor Impression Plus	Prowess v5.40
51	Definitive	Mon-Fri	16	3	48	100	3 (1 dorsal, 2 laterals)	sternal	Siemens Oncor Impression Plus	Prowess v5.51
52	Definitive	Mon-Fri	15	3	45	NA	5	Sternal	Elekta Precise	Oncentra
53	Definitive	Mon-Wed-Fri	12	3.5	42	NA	5	Sternal	Elekta Precise	Oncentra
54	Definitive	Mon-Fri	15	3	45	NA	6	Sternal	Elekta Precise	Oncentra
55	Definitive	Mon-Fri	15	3	45	NA	5	Sternal	Elekta Precise	Oncentra
56	Definitive	Mon-Fri	15	3	45	NA	5	Sternal	Elekta Precise	Oncentra
57	Definitive	Mon-Fri	15	3	45	NA	5	Sternal	Elekta Precise	Oncentra
58	Definitive	Mon-Fri	20	2.5	50	NA	6	Sternal	Elekta Precise	Oncentra
59	Definitive	Mon-Wed-Fri	12	3.75	45	NA	5	Sternal	Elekta Precise	Oncentra
60	Definitive	Mon-Wed-Fri	15	2.85	42.75	NA	6	Sternal	Elekta Precise	Oncentra
61	Definitive	Mon-Fri	15	3	45	NA	5	Sternal	Elekta Precise	Oncentra
62	Definitive	Mon-Wedn-Fri	12	3.5	42	NA	5	Sternal	Elekta Precise	Oncentra
63	Definitive	Mon-Fri	15	3	45	NA	4	Sternal	Elekta Precise	Oncentra
64	Definitive	Mon-Fri	15	3	48	100	3 (1 dorsal, 2 laterals)	NA	Varian 600C	Oncentra
65	Definitive	Mon-Fri	15	3	48	100	3 (1 dorsal, 2 laterals)	NA	Varian 600C	Oncentra
66	Palliative	Once weekly	5	1 dose 5Gy, 4 doses 8Gy	37		NA	NA	Varian 600C	Oncentra
67	Palliative	Once weekly	5	1 dose 5Gy, 4 doses 8Gy	37	200	4 (dorsal, ventral, 2 laterals)	NA	Varian 600C	Oncentra
68	Palliative	Once weekly	5	3	48	200	4 (dorsal, ventral, 2 laterals)	NA	Varian 600C	Oncentra
69	Definitive	Mon-Fri	16	2.75	42.5	91.6	3 (1 dorsal, 2 laterals)	NA	Varian 600C	Oncentra
70	Definitive	Mon-Fri	16	3	48	75	4 (1 dorsal, 1 ventral, 2 laterals)	NA	Varian 600C	Oncentra
71	Definitive	Mon-Wed-Fri	16	3	48	NA	3 (1 dorsal, 2 laterals)	NA	Varian 600C	Oncentra
72	Definitive	Mon-Fri	16	2.81	44.69	70.25	4 (1 dorsal, 1 ventral, 2 laterals)	NA	Varian 600C	Oncentra
73	Palliative	Once weekly	5	7	35	210 dorsal, 245 R and L	3 (1 dorsal, 2 laterals)	NA	Varian 600C	Oncentra
74	Definitive	Mon-Fri	15	3	45	52.5 dorsal, 90 ventral, 78.75 R and L	4 (1 dorsal, 1 ventral, 2 laterals)	NA	Varian 600C	Oncentra
75	Definitive	Mon-Fri	16	3	48	75	4 (1 dorsal, 1 ventral, 2 laterals)	NA	Varian 600C	Oncentra
76	Definitive	Mon-Fri	16	3	48	75	4 (1 dorsal, 1 ventral, 2 laterals)	NA	Varian 600C	Oncentra
77	Definitive	Mon-Fri	15	3	48	75	4 (1 dorsal, 1 ventral, 2 laterals)	NA	Varian 600C	Oncentra
78	Definitive	Mon-Wed-Fri	16	3	48	NA	3 (1 dorsal, 2 laterals)	NA	Varian 600C	Oncentra
79	Definitive	Mon-Fri	16	NA	37	75	4 (1 dorsal, 1ventral, 2 laterals)	NA	Varian 600C	Oncentra
80	Palliative	Once weekly	5	1 dose 5Gy, 4 doses 8Gy	NA	NA	NA	NA	Varian 600C	Oncentra
81	Definitive	Mon-Fri	14	NA	NA	NA	NA	NA	Varian 600C	Oncentra
82	Definitive	Mon-Fri	16	3	48	75	4 (1 dorsal, 1 ventral, 2 laterals)	NA	Varian 600C	Oncentra
83	Definitive	Mon-Fri	16	2.75	44	91.7	3 (1 dorsal, 2 laterals)	NA	Varian 600C	Oncentra
84	Definitive	Mon-Wed-Fri	15	3	45	100	3 (1 dorsal, 2 laterals)	NA	Varian 600C	Oncentra
85	Definitive	Mon-Fri	15	3	45	75	4 (1 dorsal, 1 ventral, 2 laterals)	NA	Varian 600C	Oncentra
86	Definitive	Mon-Fri	16	2.75	44	73 dorsal, 101 R and L	3 (1 dorsal, 2 laterals)	NA	Varian 600C	Oncentra
87	Definitive	Mon-Fri	16	3Gy for 8 treatments, 2.25Gy for 1 treatment, 3.1Gy for 7 treatments	48	75	4 (1 dorsal, 1 ventral, 2 laterals)	NA	Varian 600C	Oncentra
88	Palliative	Once weekly	5	6	30	150	4 (1 dorsal, 1 ventral, 2 laterals)	NA	Varian 600C	Oncentra
89	Definitive	Mon-Wed-Fri	12	4	48	100	4 (1 dorsal, 1 ventral, 2 laterals)	NA	Varian 600C	Oncentra
90	Definitive	Mon-Fri	16	3	48	75	4 (1 dorsal, 1 ventral, 2 laterals)	NA	Varian 600C	Oncentra
91	Definitive	Mon-Fri	16	3	48	NA	3 (1 dorsal, 2 laterals)	NA	Varian 600C	Oncentra
92	Definitive	Mon-Wed-Fri	16	3	48	NA	3 (1 dorsal, 2 laterals)	NA	Varian 600C	Oncentra
93	Definitive	Mon-Fri	16	3	48	113 dorsal, 127 L and R	3 (1 dorsal, 2 laterals)	NA	Varian 600C	Oncentra
94	Definitive	Mon-Fri	16	3	48	75	4 (1 dorsal, 1 ventral, 2 laterals)	NA	Varian 600C	Oncentra

LEGEND

Mon-Fri: definitive protocol where RT was delivered from Monday to Friday.
Mon-Wed-Fri: definitive protocol where RT was. delivered on Monday, Wednesday and Friday only.
NA: not available. This parameter was not available for review retrospectively.
ARPS: Addenbrooke's Radiotherapy Planning System.
L: left.
R: right.
Dorsal: dorso-ventral beam.
Ventral: ventro-dorsal beam.
Lateral: latero-lateral beam.
Anterior: cranio-caudal beam.
